# Comprehensive Geriatric Assessment as a Versatile Tool to Enhance the Care of the Older Person Diagnosed with Cancer

**DOI:** 10.3390/geriatrics4020039

**Published:** 2019-06-24

**Authors:** Janine Overcash, Nikki Ford, Elizabeth Kress, Caitlin Ubbing, Nicole Williams

**Affiliations:** 1 The College of Nursing, The Ohio State University, 1585 Neil Ave, Newton Hall, Columbus, OH 43201, USA; 2 Stephanie Spielman Comprehensive Breast Center, The Ohio State University, 1145 Olentangy River Road, Columbus, OH 43121, USA; Nikki.Ford@osumc.edu (N.F.); Eizabeth.Kress@osumc.edu (E.K.); Caitlin.Ubbing@osumc.edu (C.U.); Nicole.Williams@osumc.edu (N.W.)

**Keywords:** comprehensive geriatric assessment, CGA, multidisciplinary team, senior adult, cancer

## Abstract

The comprehensive geriatric assessment (CGA) is a versatile tool for the care of the older person diagnosed with cancer. The purpose of this article is to detail how a CGA can be tailored to Ambulatory Geriatric Oncology Programs (AGOPs) in academic cancer centers and to community oncology practices with varying levels of resources. The Society for International Oncology in Geriatrics (SIOG) recommends CGA as a foundation for treatment planning and decision-making for the older person receiving care for a malignancy. A CGA is often administered by a multidisciplinary team (MDT) composed of professionals who provide geriatric-focused cancer care. CGA can be used as a one-time consult for surgery, chemotherapy, or radiation therapy providers to predict treatment tolerance or as an ongoing part of patient care to manage malignant and non-malignant issues. Administrative support and proactive infrastructure planning to address scheduling, referrals, and provider communication are critical to the effectiveness of the CGA.

Caring for the older adult who is diagnosed with cancer can be a complex orchestration of managing existing comorbid conditions, cancer care, caregiver concerns, while maintaining quality of life [1,2,3,4]. Older people have unique healthcare needs compared to younger adults who may not have challenges regarding comorbidities [4,5,6,7], functional ability [8], transportation and social support [9]. Many academic and community cancer centers establish some type of multidisciplinary geriatric oncology program to meet the needs of the older person [10,11,12,13,14,15]. The central element associated with a geriatric oncology program is a comprehensive geriatric assessment (CGA). Despite the evidence showing the benefits of CGA, only 9% and 8% of Phase II and Phase III clinical trials use CGA [16]. Many healthcare settings do not use CGA also because of time constraints, availability of a multidisciplinary team, and lack of professionals trained in geriatrics/gerontology. Conducting a CGA is feasible in ambulatory geriatric oncology programs (AGOPs) [10,17] including radiation therapy and surgical oncology [18,19,20]. There are strategies to reduce the time and resources often required to conduct a CGA. The purpose of this article is to illustrate how CGA can be used in different types of AGOPs and is a feasible option despite limited time and personnel. A review of the classic and current literature was conducted using the Ohio State University (OSU) Health Sciences Library (HSL) including PubMed and Cumulative Index to Nursing and Allied Health Literature (CINAHL) to support this article. 

## 1. Defining a Comprehensive Geriatric Assessment

A CGA is a battery of screening tools necessary to uncover actual and potential limitations that can compromise cancer diagnosis and treatment [21]. The Society for International Oncology in Geriatrics (SIOG) recommends a CGA be administered to older patients who are receiving cancer care [22,23]. Benefits of a CGA are prolonged survival [24], prediction of those who may not benefit from treatment [25], prediction of mortality [26], of cancer treatment tolerance [27], of chemotherapy toxicities [28], of surgical complications [29], and aid in decision-making to help avoid over- and undertreatment of cancer [30]. The battery of screening tools is generally assembled to address common problems associated with aging; however, any number of valid and reliable clinical instruments can be included, depending on the resources. Some cancer centers may be able to conduct large-scale CGA with a robust multidisciplinary team (MDT), and others may limit assessment instruments and MDT members.

The comprehensive character of geriatric assessment allows clinicians to gain perspective beyond the traditional oncology-related history and physical exam [31]. A CGA can detect previously unidentified problems in approximately 70% of patients [32], which can impact cancer treatment [19] and provide the foundation for a treatment plan to address malignant and nonmalignant conditions [33]. CGA is used to develop and refine a cancer management plan specific to the needs of the person diagnosed with cancer [20]. A prime goal of geriatric oncology is helping an older person achieve the best health possible while receiving cancer care to maintain independence [4]. 

## 2. Instruments Included in a Comprehensive Geriatric Assessment

Generally, screening tools to detect depression [34], comorbidity [35], cognitive impairment [36], functional status [37,38], risk for falls [39], and nutritional status [40] are commonly included in a CGA. The CGA is multidimensional in that many types of screening instruments can be included to meet the needs of people who are diagnosed with cancer or who are receiving end-of-life care [41], caregivers [42], and providers [43]. SIOG recommends a variety of instruments that can be tailored to any patient population [44]. 

When choosing instruments to include in a CGA, consider that there are performance-based evaluations and self-report measures. Performance-based evaluations provide a depiction of a person’s capability using tools such as the Timed Up and Go Test (TUAGT) [45], balance testing [46], grip strength [47], sit-to-stand test [48], cognitive screening using the Clock Drawing Test [49], and other empirically measured tests. Self-report measures are also commonly included in the CGA, such as the Geriatric Depression Scale (GDS) [34], Activities of Daily Living Scale [37], Instrumental Activities of Daily Living [38], quality-of-life measures [50], and nutritional assessment [40]. Self-report measures tend to be rather easy to use and have validity and reliability metrics for clinical and research use. Including both self-report and performance-based evaluations provides patient perception of functioning at home in conjunction with an objective assessment. Some patients may tend to over-estimate their functional ability, and the empirical observation of task performance may help providers develop realistic management plans. 

Supporting the caregiver is also important to the health of the person with cancer [51]. The Modified Caregiver Strain Index [52,53] is a 13-item tool that measures the financial, psychological, personal, physical, and social domains of caregiving which can be incorporated into a CGA. Caregivers of people diagnosed with cancer who have functional impairment [54] and have increased comorbidity [55] report greater strain and burden. CGA can stratify people with cancer into levels of caregiver burden risk so that clinicians can recognize caregivers who may need help [42]. Caregivers of people with advanced cancer often neglect their own health and wellness and report high levels of depression and anxiety [56]. Depression is not rare among caregivers (42%), and clinicians must support and encourage health maintenance and wellness [57]. If caregiver health is not maintained and perceptions of strain and burden exist, the individual with cancer is at risk for re-hospitalization [58] and increased morbidity/mortality [59]. Help for caregivers navigating community resources, Medicare, insurance, and cancer treatment can be very welcomed [60]. Cancer can be overwhelming and expensive, and providing psychosocial support can reduce caregiver stress associated with financial toxicity [61], address depression, and establish coping strategies [62]. No matter the scale of the CGA, caregiver support is important to geriatric oncology.

## 3. Multidisciplinary Team 

A MDT has historically been used in geriatrics to administer the CGA and manage the many interwoven concerns that can affect older people [63,64]. An MDT can be composed of physicians, social workers, pharmacists, nurses, nurse practitioners, dietitians, physical therapists, and other types of healthcare professionals. Not every clinic may have access to a variety of specialists, and it is important to remember that geriatric care and screening can be provided by physicians, nurse practitioners, and nurses. An MDT may simply include a physician and a nurse who are trained in geriatrics. Administering and coordinating a CGA is well within the scope of practice of nursing and can be central to the effectiveness of the MDT [65]. 

Whatever the size, an MDT functions symbiotically to assess, manage, and monitor many limitations and complications associated with aging and deconditioning [66]. Geriatric oncology has adopted the MDT approach to improve or maintain independence [67] and to provide CGA by which to impact the cancer management plan [20]. Key to an effective MDT are communication, collaboration, and coordination [68]. A social worker, nurse practitioner, and dietitian can evaluate a patient simultaneously and hear the responses from individual assessments, so that questions are not duplicated. This method requires a cohesive teamwork, does save some time, and enhances communication within the MDT. An MDT with a perception of cohesive teamwork provides higher quality of care and less attrition in the nursing staff [69]. Communication with primary care providers and other specialists is critical to geriatric oncology and successful interventions [70]. When primary care providers and oncology providers agree on recommendations, adherence to CGA recommendations is more likely to occur [71]. 

For providers who lack a MDT, nurse-conducted CGA is a viable option. Nurses and/or advanced practice nurses often function in the role of coordinator, provider, communicator, and organizer. Awareness of the current knowledge in normative aging, geriatric syndromes, wellness, and prevention are components of nursing best practices [72]. Best practices in geriatric/gerontological competencies are provided by the American Association of Colleges of Nursing (AACN) for advanced practice and baccalaureate nurses and largely guide curriculum development for colleges of nursing throughout the United States [73,74]. However, geriatric training is often lacking in nursing schools throughout the country [75], and geriatric education is often received outside of the academic curriculum. The National Hartford Center of Gerontological Nursing Excellence (NHCGNE) aims to enhance gerontological education among nurses in the academic and clinical workforce [76]. The NHCGNE recognizes gerontological nurse educators as ***Distinguished Educators in Gerontological Nursing Program*** for working with faculty to enhance university and college curricula, educate nursing students at all levels, and work with other providers to better care for the older person [77]. It is important that nurses are educated in gerontology/geriatrics so they are prepared to assess and contribute to the care of the older person who is diagnosed with cancer [78]. 

## 4. Management of Problems Detected by Comprehensive Geriatric Assessment

Geriatric syndromes (poor functional status, cognitive impairment, frailty), life expectancy, and comorbidity are realities that oncology providers must consider when caring for older individuals. The mean number of geriatric syndromes is 2.9 in community-dwelling older people [79] and when uncontrolled, may interfere with cancer treatment. Complex problems associated with geriatric syndromes often cannot be addressed in one clinic visit or with a single medication or intervention. For frailer people, determining the cause of a problem may require an MDT-administered CGA and several clinic visits to detect and manage complex problems [80]. Good general health and absence of severe comorbidity allow older people to be considered for surgical [81] and other types of standard treatments [82].

People who have well-managed comorbidities may not have any deterioration in their functional status or life expectancy. In non-metastatic prostate cancer patients receiving treatment, 10-year life expectancy was not impacted by comorbid conditions nor age [83]. However, data show that for every chronic condition, life expectancy decreases 1.8 years [61]. Life expectancy, comorbid conditions, and functional status are sentinel factors in geriatric oncology [84]. Functional status and not chronological age is an important consideration in cancer treatment planning for the older adult [28,85].

Initiating a CGA requires a process to manage the limitations uncovered by the evaluation, and providers should be trained on how to incorporate the MDT recommendations in the decision-making process [86]. The mean number of CGA recommendations to address the uncovered limitations ranges from seven [87] to two [88], depending on the type of patient (frail, vulnerable, or fit) [89]. A CGA performed upon an initial oncology encounter can render three interventions [90]. Patients are most likely to adhere to four or less recommendations unless they present cognitive decline, in which case adherence is lower [87]. 

Follow-up care is important to determine adherence to recommendations and to reassess the issues that were previously detected [91]. The problems detected in the CGA should be managed or referred and detailed in the medical record [92,93]. How often to administer the CGA depends on the degree of fitness or frailty of the patient. A primary care nurse who is trained in geriatrics can be effective in coordinating the recommendations [94]. 

## 5. Comprehensive Geriatric Assessment with Limited Resources 

**Figure 1:**
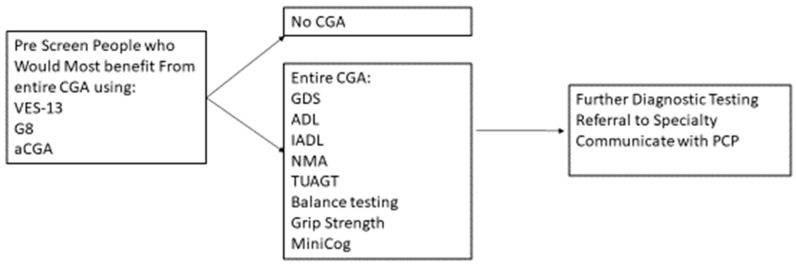
Prescreening using CGA to Determine Further Treatment or Diagnostics.

A CGA conducted by an MDT can require an hour or more to administer; however, there are strategies to conduct CGA in a timely and efficient manner. Targeting the person who would most likely benefit from the CGA with a prescreening instrument can help preserve the resources of clinical time and personnel and reduce the respondent burden (Figure 1). 

Prescreens have been developed, such as the abbreviated CGA [95], the G8 [96], and the Vulnerable Elders Survey-13 [97]. SIOG recommends several valid and reliable pre-screen tools [1]. The purpose of pre-screen tools is to target those who most benefit from conducting the entire CGA, rather than to replace a CGA. People who have functional decline and a higher risk of mortality and of cancer treatment complications tend to benefit from the CGA [98,99]. For those people who are independent and with minimal comorbid conditions, a CGA may not be as beneficial [100]. 

**Figure 2:**
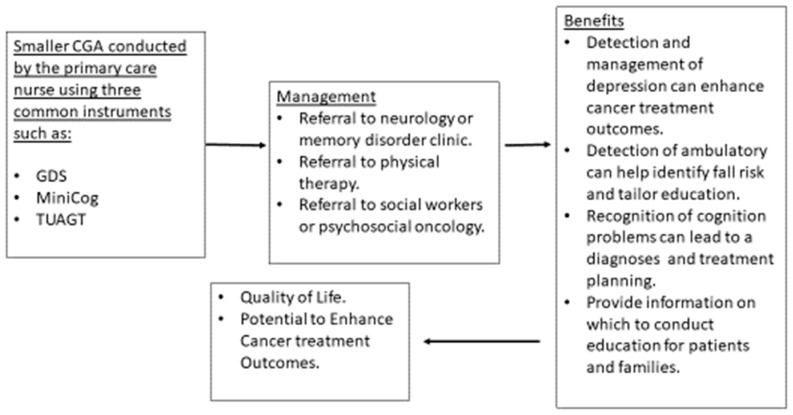
Smaller CGA to Determine Further Treatment or Diagnostics.

Depending on resources and type of healthcare setting, a CGA can be fashioned to include only several instruments rather than an exhaustive battery of tools requiring hours of clinic encounter time. Creating a smaller version of CGA which can include two or three screening instruments (GDS, Mini-Cog, TUAGT) will allow time to gain experience administering the instrument and managing the limitations. Using only two or three screening instruments has reasonable benefit to people who are diagnosed with cancer and to their families. The detection and management of depression can contribute to better cancer treatment outcomes, particularly with adherence to recommendations [101]. Benefits of screening for cognitive limitations are inconclusive [102]; however, other considerations such as planning, awareness of limitations, preparation for future and other important tasks can be very helpful for patients and families. Screening using the TUAGT can lead to physical therapy consults [88] to enhance lower extremity strength and to provide falls education and proactive planning should a fall occur (determining how to get help, keeping a phone on the bathroom floor near the bathing area). The use of three tools can provide the opportunity to address common problems that can be associated with aging without requiring the time to conduct a more robust CGA (Figure 2). 

The use of pre-screens and a smaller battery of assessment instruments is a viable option when using CGA with limited clinical resources. Understanding the versatility of CGA may motivate more clinicians to employ best practices in geriatric assessment. 

Cost and resources are a factor when establishing a geriatric oncology program; however, not all data indicate that CGA is cost-prohibitive when looking at long-term expenses and hospital stay. The SIOG suggests that CGA is cost-effective and reduces hospitalizations [103]. CGA in people who experience a hip fracture reduces hospital costs and hospital length of stay and improves health outcomes [104]. However, for those people admitted to the hospital for nonmalignant conditions, CGA is thought to slightly increase costs [105]. A Swedish study found ambulatory oncology CGA to have increased costs due to the number of interventions and increased survival [25]. Another Swedish study found ambulatory CGA to increase survival in frail people, with fewer hospital days and without higher costs [106]. In the United States, the cost savings or expenses may be different, however, people tend to benefit from CGA [85,107]. 

## 6. Models of Geriatric Oncology Programs Using CGA

AGOPs often include regular CGAs and manage a patient throughout cancer care. There are different types of AGOPs, such as those that provide ongoing geriatric oncology management, one-time consult programs, site specific programs, and programs that address patients according to age and not a particular tumor type. Scale also varies among AGOPs, with some using large MDTs and others consisting of an oncologist and a geriatric trained nurse. Regardless of the structure, AGOPs can provide CGA and offer management strategies to enhance the care of the older person diagnosed with cancer. 

The CGA can be administered by a nurse or nurse practitioner, and scores on the measures can be shared with the entire MDT, so that more in-depth screening can be conducted by the appropriate specialists. In some situations, the MDT members individually screen new patients to establish a baseline condition prior to cancer treatment. The MDT members can then evaluate the patient as needed throughout cancer treatment. Established patients who have received a baseline CGA can receive regular geriatric assessment screening every 6 months or every year. No data exist on how often to conduct a CGA; however, frail or vulnerable patients may require more frequent screening. The National Comprehensive Cancer Network (NCCN) has established guidelines for using CGA when caring for the older adult [108]. A pre-cancer treatment decision tree addresses how and when to use a prescreening and an entire CGA and how CGA can impact treatment decisions for the patient, family, and provider [108]. 

Scheduling new and established patients visits for any type of AGOP requires planning for extra time to conduct the CGA. For AGOPs conducting the entire CGA with an MDT in addition to establishing a cancer management plan, a new patient visit may require two hours. For those AGOPs using limited measures in the CGA and a limited MDT, perhaps a 30 min visit is appropriate. One-time CGA consults can be easier to schedule in that all patients tend to receive the same screening instruments and assessment from the MDT. Generally, the consult can be conducted in approximately 1.5 to 2 h per patient. Depending on the physical environment of the clinic, three patients can be scheduled every 2–2.5 hour and be accommodated with rotating members of the team conducting the assessments. 

An AGOP one-time CGA consult functions to provide recommendations for cancer treatment, identifies comorbid conditions, and addresses actual and potential risk factors that can affect health and independence. A one-time CGA consult can be helpful to surgical teams to predict complications [109] and post-surgical delirium when administered prior to surgery [110]. A one-time CGA conducted by a geriatrician prior to emergency surgery reduces hospital length of stay by 55 days [111]. Despite the positive contributions of CGA, many surgeons and other providers fail to consult geriatric services [112]. Education on the benefits of CGA in cancer treatment decision-making is critical for all cancer specialties and providers. 

Conducting a CGA and incorporating an MDT require infrastructures and administrative support to lay the foundation for a sustainable geriatric oncology. Often, facilities and providers have difficulty launching and maintaining senior adult programs, for many reasons [113]. Patient scheduling to accommodate longer visit times [114], avenues of referral when limitations are found, adapting the medical record to accommodate scores and recommendations are important tasks to address before initiating geriatric assessment [13]. AGOPs require continued evaluation and maintenance to ensure the process of clinic is working well and the MDT is functioning effectively and productively. Regular team meetings can be helpful to discuss assessment process, patients, and research activities. Regular meetings should include administration, office staff who schedule patient visits, as well as people who work with medical records, who can be helpful in establishing highly functioning clinics, especially in big medical centers. MDT meetings prior to geriatric oncology clinic are very useful to review new and established patients. 

A prime component of infrastructure is communication with other providers, which is key to the effectiveness of AGOPs. Many providers feel under-utilized in the development of cancer management plans, and communication is often poor between oncologists and primary care providers [94]. Proactive planning to establish avenues of communication to coordinate the CGA recommendations can reduce redundant assessments and increase effectiveness. Follow-up care and adherence to recommendations are likely to be improved with better communication between geriatric MDTs and other providers and typically require organizational modifications for adequate transfer of patient information [115]. 

Patient referral to an AGOP is also a consideration when establishing a clinic or a process for other oncology providers to refer patients for a one-time CGA consult or ongoing management. Awareness of the AGOP should be created within the organization and the community. Often, community members are not aware of geriatric oncology services, and providing educational symposiums or brief presentations at various sites common to potential patients and families can offer the opportunity to receive a CGA and cancer care.

An AGOP can provide valuable clinical data to enhance the care of the older person diagnosed with cancer. Establishing a research protocol incorporating CGA data can help improve the science of geriatric oncology and establish a foundation for future funding. Select CGA instruments can be useful clinically as well as appropriate for research. Dissemination is critical to geriatric oncology and helps address the importance of CGA in the care of the older person diagnosed with cancer. 

## 7. Conclusions

CGA is a versatile tool that can be integrated into various oncology clinics and specialties to provide the best care for the older person. Integrating a CGA does require administrative support, infrastructure for patient scheduling, MDT involvement, and a great deal of planning. The importance of understanding the needs of older people with cancer and of their caregivers underscores the significance of CGA and inspires a comprehensive view, helpful to make treatment decisions. CGA is the central element of geriatric oncology and the gold standard of practice to meet the needs of older people.
